# Rebaudioside A Enhances Resistance to Oxidative Stress and Extends Lifespan and Healthspan in *Caenorhabditis elegans*

**DOI:** 10.3390/antiox10020262

**Published:** 2021-02-08

**Authors:** Pan Li, Zehua Wang, Sin Man Lam, Guanghou Shui

**Affiliations:** 1 State Key Laboratory of Molecular Developmental Biology, Institute of Genetics and Developmental Biology, Chinese Academy of Sciences, Beijing 100101, China; lipan17@mails.ucas.ac.cn (P.L.); wangzh2014@genetics.ac.cn (Z.W.); 2 University of Chinese Academy of Sciences, Beijing 100049, China; 3 LipidALL Technologies Company Limited, Changzhou 213022, Jiangsu, China

**Keywords:** *C. elegans*, non-nutritive sweetener, rebaudioside A, aging, oxidative stress resistance, TOR, lipidomics

## Abstract

Non-nutritive sweeteners are widely used in food and medicines to reduce energy content without compromising flavor. Herein, we report that Rebaudioside A (Reb A), a natural, non-nutritive sweetener, can extend both the lifespan and healthspan of *C. elegans*. The beneficial effects of Reb A were principally mediated via reducing the level of cellular reactive oxygen species (ROS) in response to oxidative stress and attenuating neutral lipid accumulation with aging. Transcriptomics analysis presented maximum differential expression of genes along the target of rapamycin (TOR) signaling pathway, which was further confirmed by quantitative real-time PCR (qPCR); while lipidomics uncovered concomitant reductions in the levels of phosphatidic acids (PAs), phosphatidylinositols (PIs) and lysophosphatidylcholines (LPCs) in worms treated with Reb A. Our results suggest that Reb A attenuates aging by acting as effective cellular antioxidants and also in lowering the ectopic accumulation of neutral lipids.

## 1. Introduction

Obesity and its related spectrum of metabolic disorders have emerged as a global health epidemic in recent decades. It is widely believed that several risk factors, such as high-sugar diet or high-fat diet, partly contribute to the increasing occurrence of obesity and its related health problems, including type 2 diabetes, fatty liver disease, cardiovascular disease, and hypertension [1]. Non-nutritive sweeteners (NNSs) are increasingly used to replace traditional caloric sugars to reduce energy intake while maintaining tasting flavor in foods and beverages. NNSs are particularly useful alternative sweeteners for diabetics and individuals who need to control their blood sugar levels and minimize excessive weight gain [2]. Saccharin, aspartame, acesulfame potassium (Ace K), sucralose, neotame, rebaudioside A (Reb A), and cyclamate are food additives approved by the U.S. Food and Drug Administration (FDA) as Generally Recognized as Safe Status (GRAS) for consumption within their reasonable dose and daily intake limits [3,4,5]. The safety of NNSs, however, has remained somewhat controversial, as some of their unexpected impacts on metabolism are only beginning to unfold with recent investigation. For example, it was found that sucralose could release water and hydrogen chloride on heating to 125 °C [6], and promote the accumulation of reactive oxygen species (ROS) in mice. Sucralose also has adverse effects on glucose metabolism and gut hormones and induces intensely sweet taste preference that ultimately leads to irrational food intake [3,7,8]. Artificial NNSs application was also reported to elevate the risk of various cancers [9]. Unlike artificial NNSs, Reb A as a natural NNS has been found to possess a variety of biological functions beneficial to organisms.

Reb A is a natural product obtained from the extracts of *Stevia rebaudiana* (Bertoni), a perennial shrub in the sunflower family of Asteraceae (Compositae), which is native to the border area in Paraguay and Brazil. It was discovered that *Stevia* leaves are 30 times sweeter than sucrose, whereas stevia glycosides isolated from the leaves are 200–300 times sweeter than sucrose [10]. Stevia glycoside is also considered to be a safe natural NNS, with few side effects in animal experiments and human studies of hypertensive patients [11]. Reb A and stevioside isolated from *Stevia* share the basic structure of stevia glycosides [12,13]. Purified Reb A was approved by FDA in 2008 as GRAS. In contrast to most other NNSs, previous studies have demonstrated that Reb A is beneficial to metabolic health. For instance, Reb A was found to influence the cell cycle of human epidermal cells by increasing the distribution of cells in the S phase, while reducing that in G2/M phase [14]. Reb A also inhibits the inflammatory response in lipopolysaccharide-activated RAW264.7 mouse macrophage cells by impeding the secretion of interleukin-1α/-1β and other cytokines [15], and protects against tetrachloromethane-induced oxidative injury in human liver hepatocellular carcinoma (HepG2) cells [16]. Mice treated with long-term and low-dose Reb A were found to have altered gut microbiota composition with minimal effect on glucose metabolism and weight gain [17]. Reb A exerts anti-inflammatory, antioxidant, and antifibrotic effects on hepatic fibrosis induced by thioacetamide in rats [18]. Reb A was also found to significantly ameliorate murine non-alcoholic steatohepatitis and decrease hepatocyte triglyceride level [19], albeit the precise mechanism has remained obscured. The effect of Reb A on physiological aging, as well as on the healthspan and lifespan in model organisms, has not been previously investigated.

*Caenorhabditis elegans* (*C. elegans*) has been used as a model organism to study various biological processes, including cell polarity, cell signaling, cell cycle, gene regulation, senescence, autophagy, and metabolic processes [20]. *C. elegans* is also widely applied for rapidly screening metabolic responses to drugs or natural extracts, which provides the basis for mammalian biological experiments [21]. Moreover, worms represent an excellent model for aging studies. Their behavior and physical indices, such as pumping rate, lipofuscin accumulation, and locomotion can reflect aging rate and health status to a certain extent, facilitating investigation on the effects of different metabolites on age-related changes [22,23]. At present, several theories propose different underlying mechanisms of aging, which include the free radical damage theory, caloric restriction theory, and telomere senescence theory. The free radical aging hypothesis proposes that oxidative damages infringed upon cells and tissues may cause aging [24]. It is worth noting that reactive oxygen species (ROS) are considered to be harmful by-products from aerobic respiration, causing oxidative damage and accelerating the development of age-dependent diseases when accumulated in excess [25,26].

Lipids, especially polyunsaturated fatty acids (PUFAs), are susceptible to ROS attacks, and lipid peroxidation plays an important role in intracellular aging [27]. In addition to exerting structural functions, specific lipids, such as phosphatidylinositols (PIs) and phosphatidylinositol phosphates (PIPs), also partake in signal transduction, membrane trafficking, and other biological processes [28]. Mammalian target of rapamycin (mTOR) pathway integrates signals from growth factors and nutrients to facilitate cellular metabolic transition from catabolism to anabolism, promoting cell growth and cell cycle progression [29]. mTOR is activated in response to increases in cellular phosphatidic acids (PAs), which interact and stabilize the FK506-binding protein-12-rapamycin-binding (FRB) domains of mTOR complexes 1 (mTORC1) and mTORC2 [30].

Herein, we explored the effects of Reb A on the healthspan and lifespan of nematodes and investigated the potential mechanisms underlying Reb A-induced metabolic changes using a combination of transcriptomics and lipidomics approaches.

## 2. Materials and Methods

### 2.1. Strain and Culture Conditions

The wild-type strain N2 and *Escherichia coli* strain OP50-1 were obtained from the *Caenorhabditis* Genetics Center (CGC) at the University of Minnesota (Minneapolis, MN, USA), while mutant strains *xdEx1001* (P*daf-22*-PLIN1:GFP) were gifts from Dr Xun Huang’s laboratory (IGDB, China). Streptomycin-resistant *Escherichia coli* strain OP50-1 as a unique food source can exclude interference from other bacteria. Nematodes were cultured on nematodes growth media (NGM) with active OP50-1 as the food source in biochemical incubators at 20 °C for all experiments.

All experiments were performed on age-synchronized worms and cultured to the L4 laval stage on normal NGM [31]. Synchronized cohorts of *C. elegans* were prepared using the bleaching method (10% NaClO/ddH_2_O/10 M NaOH = 2.4:16.6:1). First, reproductive adult hermaphrodites were washed in 50 mL falcon tube using M9 buffer (5.8 g Na2HPO4, 3.0 g KH2PO4, 0.5 g NaCl, and 1.0 g NH4Cl dissolved in 1 L ddH_2_O) containing 0.005% NP40, and further washed by M9 buffer three times to remove traces of NP40 and bacteria. The resultant worms and embryos were resuspended in the bleach solution, and embryos obtained after bleaching were rinsed three times with M9 buffer. The suspension was filtered through a 40 μm cell sieve to separate embryos from worm carcasses. Embryos were transferred onto NGM plates without food for 12 h to hatch and arrest at L1 stage, and synchronized L1 larva were washed off the plates and deposited onto NGM plates with active OP50-1 bacteria to grow to the L4 stage. L4 stage worms were used for subsequent experiments.

The Reb A NGM were prepared as follow: 1.67 mM Reb A (0.32 g Reb A powder (Aladdin Chemicals Co., Shanghai, China) in 200 mL ddH_2_O) filtered with 0.2 μm cell fiter (Sartorius, Beijing, China) and stored in 4 °C prior to use. Stock solutions containing 7.5, 15, 22.5, and 30 mL of Reb A were added into 1.5 L NGM to get final concentrations of 0.0083, 0.017, 0.033, and 0.05 mM Reb A, respectively. Standard NGM was used as the control group. Both Reb A treatment group and control group used inactive OP50-1 (65 °C, 35 min) as the food source to reduce the effect of bacterial metabolism on nematode metabolism [32].

### 2.2. Pharyngeal Pumping Rate Assay

The pharyngeal pumping rate was monitored and quantified on the 5th and 10th day of adulthood, sequentially, under a stereozoom (Motic, Xiamen, China) as described previously [33]. Worms from Reb A (0.0083, 0.017, 0.033, and 0.05 mM), neotame (0.5, 1, 2, and 4 mM), and Raffiose (1, 2, 3, and 4 mM) treatment groups and a control group were evaluated. Fifteen worms from each group were randomly selected for the assay. At room temperature, the pharyngeal pumping frequency of each nematode was counted over a 20 s interval for three times, at room temperature.

### 2.3. Lipofuscin Assay

Assay of lipofuscin level in vivo was conducted, as previously described [34]. A total of 200 worms from the 0.0083 mM Reb A and the control group grown to the 8th day of adulthood were thoroughly washed with M9 buffer to remove bacteria. Worms were placed on 2% agarose pad and anesthetized with 10 mM levamisole. Images were captured by Observer Z1 laser scanning confocal microscope (Zeiss, Jena, Germany) under DAPI filter. Thirty worms from each group were captured and experiments were performed in triplicates.

### 2.4. Lifespan Assay

Hermaphrodites were crossed three times with males before lifespan assay. Approximately 200 L4 stage worms were used for the assay, as previously described [35]. Briefly, 200 worms from each group were transferred to 6 cm plates. During the reproduction period, worms were transferred to fresh plates each day to exclude hatching embryos. Worms with abnormal behaviors such as crawling off the plate, died away from the agar and abnormal phenotypes such as “bagging”, i.e., the hatching of live progeny inside the hermaphrodite, or “explosion”, i.e., the eruption of intestines from the vulva, were censored from the analysis [36]. Percent survival on each plate was recorded daily until all the worms were dead. Worms that did not respond to a mechanical stimulus was scored as dead. A Kaplan–Meier lifespan analysis was conducted, and *p* value was calculated using a log-rank test.

### 2.5. Swimming Assay

Swimming assays were performed as previously described [37]. At least 30 worms on the 5th and 10th day of adulthood were randomly selected and transferred to a glass slide containing 10 μL of M9 buffer. They were allowed to acclimate to liquid medium for 10 s, and then body swing for 30 s was scored. The experiment was repeated three times.

### 2.6. Osmotic Avoidance Assay

Osmotic avoidance assay was conducted at 20 °C, as previously described [38]. Synchronized L4 stage worms were transferred onto 9 cm NGM plates with or without 0.0083 mM Reb A treatment. On reaching Day 1 of adulthood, hermaphrodites were placed on a blank NGM with a small drop of 0.3 M glycerin solution dissolved in M9 buffer in the worm’s forward path. If the worm stopped moving forward, positive osmotic avoidance was scored. Each nematode was tested 5 times and the percent osmotic avoidance of each nematode was calculated. A total of 20 worms were tested for each group.

### 2.7. Brood Size Assay

Swimming assays were performed, as previously described [39]. Two worms at L4 stage were transferred to each 3 cm NGM plates with or without 0.0083 mM Reb A. Worms were transferred to fresh plates daily over the next 7 days, and the total number of embryos laid was counted. The experiment was repeated three times.

### 2.8. Body Length and Body Width

This assay was carried, out as previously described [40]. L4 stage worms were cultured on NGM plates with or without Reb A (0.0083, 0.017, 0.033, and 0.05 mM), and photos were taken with an Olympus MVX10 fluorescence microscope (Olympus, Beijing, China) on the first day of adulthood. Photos of 50 worms were evaluated from each group, and the experiment was carried out in triplicate.

### 2.9. Heat Stress Assay

Heat stress assay was performed, as previously described [41]. Briefly, 100 L4 stage worms were put onto 6 cm NGM plates with or without 0.0083 mM Reb A, and allowed to grow to the 5th day of adulthood at 20 °C. The temperature was shifted to 35 °C for 5 h heat shock. After heat shock, worms were allowed to recover at 20 °C for 96 h and the number of alive and dead worms were scored. Experiments were conducted in triplicate and the results were presented as the percentage of surviving nematodes.

### 2.10. Acute Oxidative Stress Assay

Acute oxidative stress assay was performed, as previously described with modification [42]. A total of 1000 worms at L4 stage were cultured on 9 cm NGM plates with or 0.0083 mM Reb A to the 6th day of adulthood at 20 °C. Worms were washed off plates with M9 buffer and about 100 worms were transferred to transparent 96-well plates containing 200 mM paraquat dissolved in double deionized water (ddH_2_O) in each well. Worms were treated for 6 h, and the lid cover was opened at 1 h intervals to supply oxygen. After 6 h, the percent survival from each group was scored.

### 2.11. Accumulation of Cellular Reactive Oxygen Species (ROS)

This assay was carried out, as previously described [43]. ROS accumulation was evaluated in worms on the 6th day of adulthood in the presence of 7 mM paraquat treatment for 2 h. At the end of treatment, 190 μL of worm suspension was added to individual wells in a 96-well black plate each containing 10 μL of 10 mM H2DCF-DA (MCE, Shanghai, China). The fluorescence intensity of each well was measured with 485 nm excitation and 535 nm emission filters at 30 min intervals for a total duration of 6 h via multifunction reader (BioTek, Winooski, VT, USA). Worms were scored under stereozoom in each well and data were normalized to worm number.

### 2.12. Food Intake Assay

This assay was carried out in liquid culture medium, as previously described [44]. Worms were cultured in 190 μL of S-complete buffer (5.8 g NaCl, 50 mL 1 M phosphate buffer solution dissolved in 924 mL ddH_2_O, and after sterilization added 3 mL 1 M MgSO_4_, 6 mL 0.5 M CaCl_2_, 6 mL 100× trace metal solution, 1 mL 5 mg/mL cholesterol, and 10 mL 1 M potassium citrate) with 50 μg/mL carbenicillin and 0.1 μg/mL fungizone in black, flat-bottom, optically clear 96-well plates. Each well contained 15 nematodes and 10 μL of 6 mg/mL OP50-1. Age-synchronized nematodes were seeded at L1 larva and grown at 20 °C. Plates were covered with sealers (Biorad, Rockville, MD, USA) to prevent evaporation. A final concentration of 12 mM 5-fluoro-2′-deoxyuridine (FUDR) was added to prevent egg laying. Reb A was added at L4 stage in the treatment group. Absorbance at 600 nm (OD600) in each well was measured using microplate spectrophotometer (EON, Winooski, VT, USA). Measurements were taken every 24 h starting from 1st of adulthood. Before measuring OD600, each plate was placed onto a plate shaker for 25 min. Living animals in each well were scored microscopically on the basis of movement on the 4th day of adulthood. For comparisons between different treatments, food intake was expressed as a percentage relative to worms in the control group.

### 2.13. RNA Extraction and Transcriptome Analysis

RNA was extracted using RNA simple Total RNA Kit (TIANGEN, Beijing, China). Briefly, 3000 worms were collected on 10th day of adulthood, snap-frozen using liquid nitrogen and stored at −80 °C. Two spoons of sterilized ceramic beads and 500 μL Trizol buffer were added to each sample and worms were homogenized on a bead ruptor (OMNI, Seattle, WA, USA), followed by adding 500 μL Trizol buffer and incubated at room temperature for 5 min. Chloroform was added for phase separation, and the colorless aqueous phase was transferred to new 1.5 mL tubes following centrifugation at 4 °C, 12000 rpm for 10 min. Anhydrous ethanol was slowly added to aqueous phase and sample suspensions were transferred to CR3 columns for RNA purification. The library construction and sequencing were performed at Shanghai Biotechnology Corporation with an Illumina HiSeq 2500 instrument (Illumina, San Diego, CA, USA). RNA integrity was assessed using the RNA Nano 6000 Assay Kit of the Agilent Bioanalyzer 2100 system (Agilent Technologies, Santa Clara, CA, USA). Gene ontology (GO) enrichment analysis of the differentially expressed genes (DEGs) was implemented by the GOseq R packages based Wallenius non-central hyper-geometric distribution [45], which can adjust for gene length bias in DEGs. KEGG [46] is a database resource for understanding high-level functions and utilities of the biological system, such as the cell, the organism, and the ecosystem, from molecular-level information, especially large-scale molecular datasets generated by genome sequencing and other high-throughput experimental technologies (http://www.genome.jp/kegg/, accessed on 25 January 2021). We used KOBAS software to test the statistical enrichment of DEGs in KEGG pathway database.

### 2.14. Postfix Nile Red (NR) Staining

Postfix Nile red (NR) staining on the 5th and 10th day of adulthood were performed, as described [47]. Worms were washed using 1 mL PBST (PBS with 0.01% Triton X-100) and centrifuged at 560× *g* for 1 min. Supernatant was discarded. Worms were washed repeatedly for three times until the supernatant turned clear. The worms were incubated with 100 μL of 40% isopropanol, and then centrifuged at 560× *g* for 1 min. Worms were incubated in darkness for 2 h in 600 μL of working solution (1 mL 100% isopropanol containing 6 μL 5 mg/mL Nile red powder). After centrifugation at 560× *g* for 1 min, supernatant was discarded and 600 μL PBST was added. The worms were incubated for 30 min to remove excess NR staining. After removing most of the staining solution, the fixed worms were mounted onto 2% agarose pads for microscopic observation and photography with Observer Z1 Laser-scanning confocal microscope (Zeiss, Jena, Germany). Image J software was used to split picture to three color channels, and relative fluorescence intensity under the red channel was calculated.

### 2.15. Oxygen Consumption Rate (OCR) Measurement

The oxygen consumption rate (OCR) measurement was carried out, as previously described [48]. We used Seahorse Xfe96 (Agilent, Santa Clara, CA, USA) to detect 10th day adult OCR under normal culture condition and under oxidative stress. Briefly, after hydrating the probe and system equilibration, we put 3–20 worms into each sample wells for OCR measurement. The results obtained were normalized to worm number in each well to reflect the worm mean OCR.

### 2.16. Malondialdehyde (MDA) Content Assay

The malondialdehyde (MDA) content was quantitated by MDA kit (Solarbio, Beijing, China) using worms on the 6th day of adulthood after exposure to oxidative stress induced by 7 mM paraquat. Sample absorbance at 450, 532, and 600 nm were measured using multifunctional reader (BioTek, VT, USA). Piece bicinchoninic acid (BCA) (Thermo Fisher, Waltham, MA, USA) kit was used to quantify MDA content.

### 2.17. Lipid Extraction and MS Analysis

Lipids were extracted from 10,000 worms on the 10th day of adulthood, as previously described using a modified version of the Bligh and Dyer’s protocol [49]. Lipidomics analyses were conducted on an 1260 HPLC (Agilent, CA, USA) coupled with a QTRAP 5500 (Sciex, Framingham, MA, USA), as previously described [50,51]. Lipids were quantitated by referencing to spiked internal standards, which included PA-34:0(17:0/17:0), PC-d_31_-34:1(16:0/18:1), PE-d_31_-34:1(16:0/18:1), PG-d_31_-34:1(16:0/18:1), d_7_-PI33:1(15:0/18:1), PS-d_31_-34:1(16:0/18:1), LPC-d_4_-26:0, LPE-C17:1, LPG C17:1, LPI C17:1, LPS-C17:1, CL 22:1(3)/14:1, DAG 16:0/16:0-d_5_, DAG 18:1/18:1-d_5_ from Polar Lipids (Avanti, AL, USA) and TAG (16:0)_3_-d_5_, TAG (14:0)_3_-d_5_, TAG (15:0)_3_-d_29_ from isotopes (CDN, Montreal, QC, Canada).

### 2.18. Statistical Analysis

All results are presented as mean ± SEM, and statistical analyses were performed using SPSS software 24.0 (SPSS Inc., Chicago, IL, USA) and presented by GraphPad Prism 7.0 software (GraphPad software, San Diego, CA, USA). For two group comparison, unpaired two tailed *t*-test was applied. Multiple group comparisons were conducted using one-way analysis of variance (ANOVA), and pairwise statistical significance was evaluated by Dunnet’s test.

## 3. Results

### 3.1. Reb A Extends Lifespan in C. elegans

**Figure 1:**
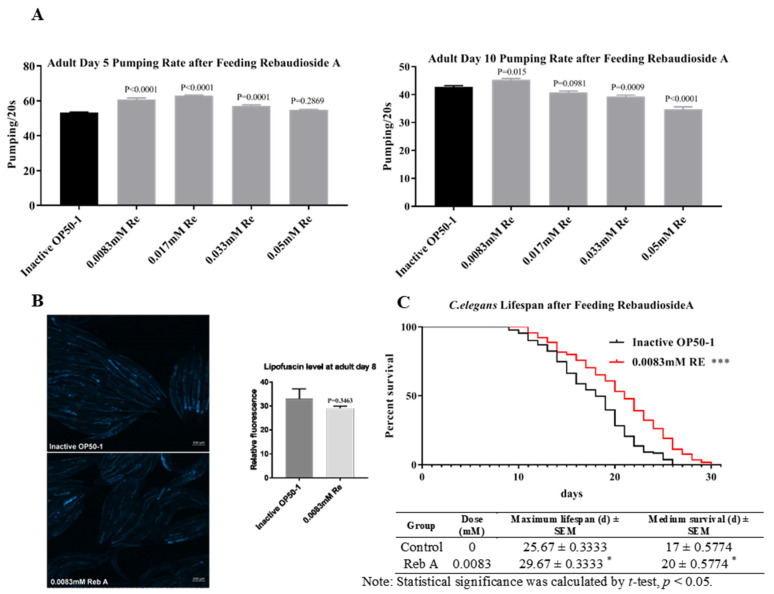
Reb A extends lifespan in *C. elegans*. (**A**) Pumping rate in worms on the 5th and 10th day of adulthood in wild-type N2 strain on both control nematodes growth media (NGM) and NGM supplemented with rebaudioside A (Reb A) at designated concentrations (0.0083, 0.017, 0.033, and 0.05 mM). One-way ANOVA was used for multiple group comparisons and pairwise significance was evaluated by Dunnet’s test; (**B**) Lipofuscin fluorescence in worms on the 8th day of adulthood grown under control NGM and 0.0083 mM Reb A NGM. Statistical significance was calculated by unpaired *t*-test; (**C**) Survival plot and lifespan assay comparing worms grown on control NGM and that supplemented with 0.0083 mM Reb A. Statistical significance was calculated by log-rank (Mantel–Cox) test, * *p* < 0.05, *** *p* < 0.001. Maximum and medium survival were shown in the table appended below.

*C. elegans* exhibits several changes in physiological phenotypes during senescence. For instance, pharyngeal pumping rate and lipofuscin accumulation are well-known hallmarks of aging for *C. elegans*. Pharyngeal pumping rate increases gradually throughout the developmental larval stages and peaks at the L4 stage, and then decreases gradually during aging [52]. The accumulation of lipofuscin, which comprises highly oxidized proteins and lipids, is also observed with aging [22]. Preliminary screening of a few sweeteners demonstrated the potential of Reb A with regard to anti-aging, while treatment with neotame or low caloric sweetener raffinose exerted adverse effects on nematodes (Figure S1). On the fifth day of adulthood, Reb A (0.0083, 0.017, and 0.033 mM) treated worms showed significantly elevated pharyngeal pumping rate, whereas in 10-day-old adults, only worms treated with 0.0083 mM Reb A exhibited increased pharyngeal pumping rates relative to the control (Figure 1A). In addition, a preliminary investigation using various concentrations of Reb A indicated that 0.0083 mM Reb A treatment prolonged the lifespan of nematodes to the greatest extent (Figure S2). Therefore, we had subsequently chosen to investigate the metabolic effects of treating worms with 0.0083 mM Reb A. We further examined in vivo lipofuscin accumulation with aging by detecting blue autofluorescence (via a DAPI filter) but observed no significant changes in lipofuscin autofluorescence in 8-day-old adults between treatment and control groups (Figure 1B). The results from the lifespan assay indicated that supplementation with 0.0083 mM Reb A significantly extended longevity (*p* < 0.001), maximal lifespan (15.58% increase), and medium lifespan (17.6% extension) in *C. elegans* as compared with the control medium (Figure 1C). These results revealed that Reb A can delay aging and extend longevity in *C. elegans*.

### 3.2. Reb A Extends Healthspan in C. elegans

**Figure 2:**
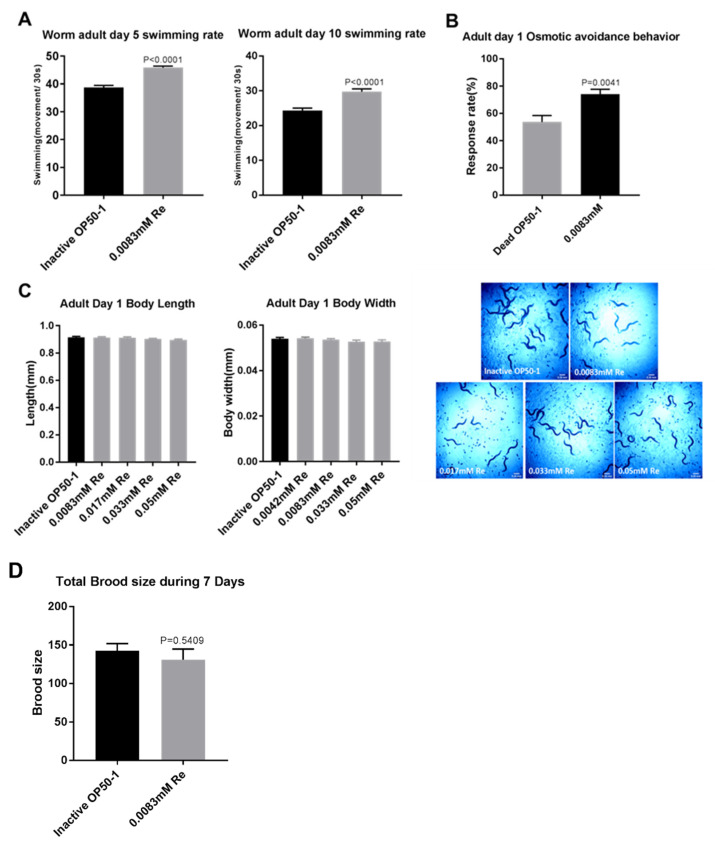
Reb A extends healthspan in *C. elegans*. (**A**) Swimming movement in *C. elegans* on the 5th and the 10th day of adulthood on control NGM and NGM supplemented with 0.0083 mM Reb A; (**B**) Osmotic avoidance behavior in *C. elegans* on the 1st day of adulthood on control NGM and NGM supplemented with 0.0083 mM Reb A. Statistical significance was calculated by unpaired *t*-test; (**C**) Body length and width of *C. elegans* on the 1st day of adulthood grown on control NGM and NGM supplemented with different concentrations of Reb A. Statistical significance was calculated by Dunnet’s test; (**D**) A comparison on total brood size over a seven-day reproductive period.

In addition to lifespan, healthspan has emerged as an increasingly important parameter for evaluation of anti-aging capacity [53]. Thus, we also detected additional physical parameters, including swimming behavior and osmotic avoidance behavior, which are effective indicators of healthspan in *C. elegans*. Swimming behavior was assessed via measuring the number of body swings in nematode in liquid medium. *C. elegans* treated with 0.0083 mM Reb A exhibited enhanced swimming behavior (Figure 2A); 0.0083 mM of Reb A supplementation elevated swimming ability in young *C. elegans* (5-day-old adults), and also in aged worms (10-day-old adults). Thus, Reb A significantly delayed deterioration in swimming movement caused by aging. Osmotic avoidance behavior is regulated by multidirectional induction ash sensory neurons (ASH), which mediate evasion from hypertonic solutions as well as other noxious chemicals and mechanical stimuli [37,54]. Our result showed that supplementation with 0.0083 mM Reb A significantly improved osmotic avoidance behavior (Figure 2B), which implied that Reb A enhances ASH sensitivity. However, Reb A supplementation had no significant effects on brood size, body length, and body width (Figure 2C,D). Therefore, the above results showed that Reb A supplementation increased pharyngeal pumping, swimming movement, and osmotic avoidance behavior, which indicated the maintenance of healthspan in *C. elegans*.

### 3.3. Reb A Enhances Resistance to Oxidative Stress in C. elegans

**Figure 3:**
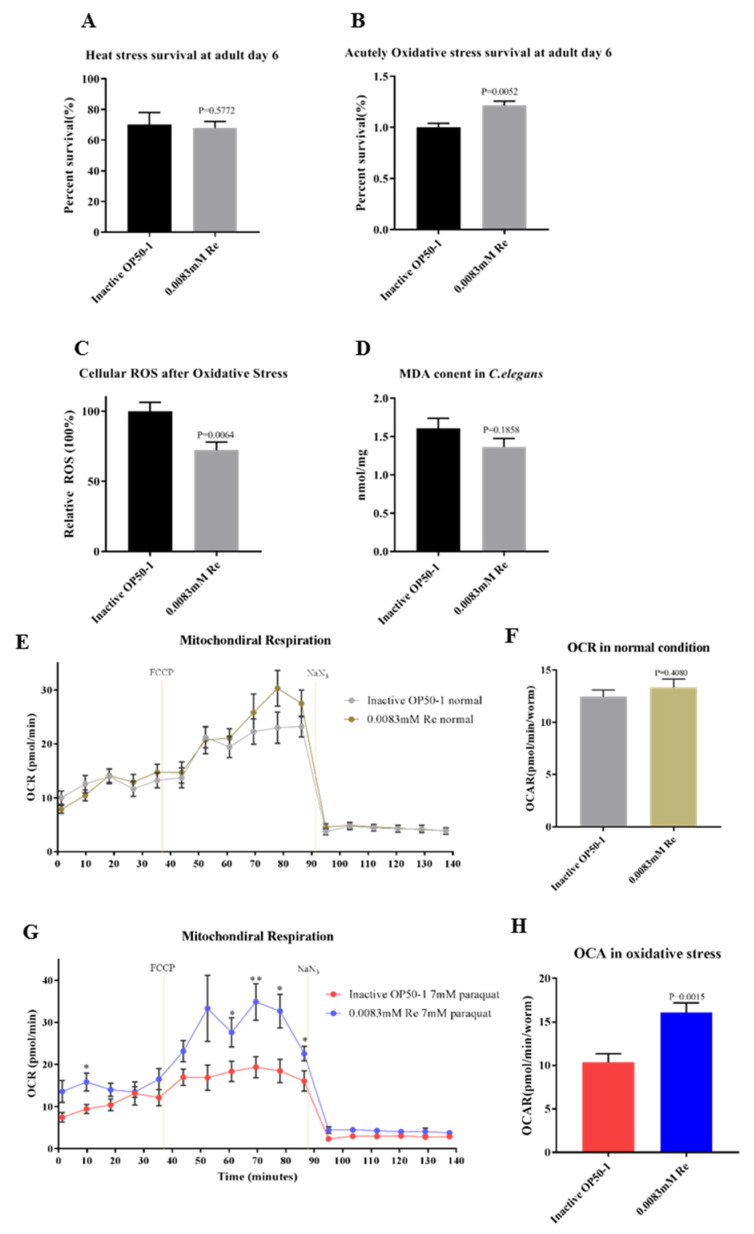
Reb A enhances resistance to oxidative stress in *C. elegans*. (**A**) Heat stress survival in the 6-day-old adult worms from Reb A and control groups; (**B**) Acute oxidative stress (treatment with 200 mM paraquat for 2 h) survival in 6-day-old adult worms from Reb A and control groups; (**C**) Relative cellular ROS level after treatment with 7 mM paraquat in the 6-day-old adult worms from Reb A and control groups; (**D**) MDA content in the 6-day-old adult worms from Reb A and control groups following oxidative stress exposure; (**E**) Under normal condition, basal respiration, maximal respiration, and spare capacity of 0.0083 mM Reb A-treated 6-day-old adults were not significantly different from those of the control group (*n* = 16 wells); (**F**) OCR was not statistically different between the 6-day-old adult worms treated with 0.0083 mM Reb A and the control group; (**G**) The 6-day-old adult worms from the 0.0083 mM Reb A group showed increased basal respiration and maximal respiration after 2 h of 7 mM paraquat treatment as compared with the control group (*n* = 12 wells), * *p* < 0.0021, ** *p* < 0.0332; (**H**) OCR under oxidative stress was significantly increased in 6-day-old adult worms treated with 0.0083 mM Reb A as compared with the control group. Statistical significance was calculated by unpaired *t*-test.

As lifespan extension is closely associated with enhanced survival under exposure to different stressors [55], next, we examined whether Reb A supplementation altered the capacity of worms to withstand heat stress and oxidative stress. After exposing 5-day-old adult worms to heat stress (i.e., 35 °C for 5 h) and following a recovery period at 20 °C for 96 h, there was no obvious difference in survival rates between Reb A-treated group and control group (Figure 3A). Next, we examined the effect of Reb A treatment on oxidative stress resistance. Worms were exposed for 6 h to 200 mM paraquat, an intracellular free radical-generating compound that induces acute oxidative stress [36]. Significant increase in survival rate was observed in the 0.0083 mM Reb A treatment group (Figure 3B). Therefore, while Reb A treatment had no effect on the resistance to heat shock, Reb A alleviates acute oxidative stress induced by paraquat in *C. elegans*.

ROS are highly reactive oxygen molecules that induce genotoxicity and physiological damages that destroy the innate mechanisms of stress resistance, which may lead to DNA damages, altered gene expression, perturbed cellular signaling, lipid peroxidation, and imbalances in protein homeostasis, ultimately resulting in cell senescence and death [41]. Excessive accumulation of ROS is also associated with aging and a myriad of aging-related diseases, including cardiovascular diseases and cancers. Therefore, we postulated that Reb A-enhanced resistance to oxidative stress may be attributed to a higher capacity to cope with intracellular ROS. A common fluorescent probe H_2_DCFDA was used to quantitate intracellular ROS. It was noted that 0.0083 mM Reb A supplementation significantly reduced cellular ROS following exposure to oxidative stress (i.e., 7 mM paraquat for 2 h) in 6-day-old adult worms as compared with the control group (Figure 3C). Malonaldehyde (MDA), one of the end products of lipid peroxidation triggered by ROS, is an important biomarker of cellular oxidative stress [56]. We further examined the influence of Reb A supplementation on MDA content in worms treated with 7 mM paraquat (Figure 3D). MDA content was slightly reduced in Reb A-treated worms but failed to reach statistical significance (*p* = 0.1858). Thus, these results showed that Reb A supplementation at 0.0083 mM resulted in increased resistance to oxidative stress induced by paraquat treatment, mainly attributed to reductions in cellular ROS production upon oxidative stress exposure.

The mitochondrial free radical theory of aging suggests that the efficiency of the respiratory chain decreases with aging, accompanied by increasing electron leakage from the respiratory chain and greater production ROS production. Excess accumulation of ROS also causes mitochondrial dysfunction [57]. In addition, mitochondrial respiration is decreased gradually during aging in *C. elegans*. We observed no significant difference in OCR values between the 0.0083 mM Reb A group and the control group under non-stressed conditions (Figure 3E,F). As Reb A was shown to reduce ROS accumulation under oxidative stress, we utilized Seahorse XFe96 to measure OCR under oxidative stress in Reb A-treated and control groups. The results showed that 0.0083 mM Reb A treatment significantly increase OCR under oxidative stress, suggesting the protective effects of Reb A on mitochondrial respiration (Figure 3G,H).

### 3.4. Reb A Inhibited the CeTOR Signaling Pathway

**Figure 4:**
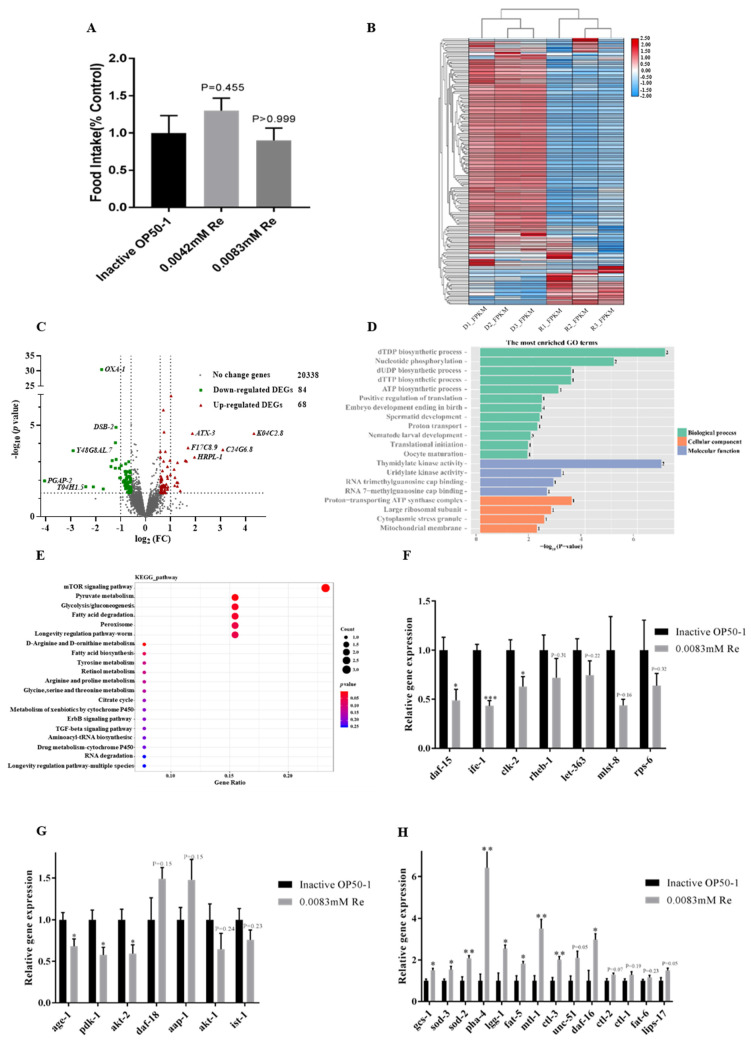
Reb A inhibited CeTOR signaling pathway. (**A**) Food intake showed no significant difference in worms grown under control NGM and NGM supplemented with both 0.0042 mM and 0.0083 mM Reb A; (**B**) Bidirectional clustering heat map of differentially expressed genes (DEGs) expression based on FPKM data of RNA-seq transcriptome; (**C**) Volcano plot illustrates DEGs in Reb A treatment groups relative to the control group. Red dots are upregulated genes while green dots were downregulated genes; (**D**) Top 20 GO terms based on transcriptome of Reb A-treated worms relative to controls. The vertical axis corresponds to the GO terms divided into different categories, and the horizontal axis displays the value of -log_10_ (*p*-value). The number on the right of each GO term indicates the number of DEGs. Green represents biological processes, red represents cellular components, and blue represents molecular functions; (**E**) Top altered pathways with Reb A treatment in *C. elegans*. The vertical axis corresponds to the KEGG pathways, and the horizontal axis displays the enriched value expressed as the ratio of DEG to the total gene number in each pathway. The size and color of dots indicates the DEG gene number and the *p*-value, respectively; (**F**) Expression level of genes in CeTOR signaling pathway, which include *daf-15*, *ife-1*, *clk-2*, *rheb-1*, *let-363*, *mlst-8*, and *rps-6*; (**G**) Gene expression levels in PI3K/Akt signaling pathway, and selected genes include *age-1*, *pdk-1*, *akt-2*, *daf-18*, *aap-1*, *akt-1*, and *ist-1*; (**H**) The expression levels of genes related to longevity signaling pathways of nematode include *gcs-1*, *sod-3*, *sod-2*, *pha-4*, *lgg-1*, *fat-5*, *mtl-1*, *ctl-3*, *unc-51*, *daf-16*, *ctl-2*, *fat-6*, and *lips-17*. Unpaired two-tailed test was used to examination of statistical significance, *p* value represented by GP type, *** *p* < 0.0002, ** *p* < 0.0021, * *p* < 0.0332, and others showed precise *p* values.

We showed that Reb A treatment is anti-aging and brings forth longevity extension and higher resistance against oxidative stress in *C. elegans*. However, the mechanism by which Reb A extends lifespan in nematodes remains unclear. Dietary restriction (DR) was previously found to significantly increase longevity and healthspan in yeast, nematodes, and mammals [37]. To confirm whether Reb A supplementation extends lifespan mediated by DR, we analyzed food intake of nematodes in liquid culture. Reb A treatment started from L4 larval stage to the fourth day of adulthood, and OD600 value of the culture medium was measured by spectrophotometer. There was no significant difference in food intake among 0.0083 mM Reb A group (*p* = 0.9478), 0.0042 mM Reb A group (*p* = 0.7740), and control groups (Figure 4A), which indicated that Reb A supplementation prolongs lifespan via DR-independent mechanism.

Next, we investigated changes in transcriptomes between the 10-day-old adult worms grown under control NGM and NGM supplemented with 0.0083 mM Reb A. Using fold change ≥1.5 and *t*-test *p*-values <0.05 as thresholds to select differentially expressed genes (DEGs) among 20,490 genes profiled between the treatment and control groups, we found 152 DEGs, which include 68 upregulated DEGs, and 84 downregulated DEGs in the Reb A supplemented group relative to the control group (Figure 4B,C). Bidirectional clustering analysis of DEGs based on the fragments per kilobase million (FPKM) values showed maximum horizontal distance between the control group and the Reb A treatment group (Figure 4B).

The DEG annotation analysis is helpful for the interpretation of gene functions. Therefore, DEGs with corrected *p* < 0.05 were used for GO function annotation and KEGG pathway analysis. In this study, among the top 20 enriched GO terms, 12 biological processes were represented, including dTDP biosynthetic process, nucleotide phosphorylation, and dTTP biosynthetic process, and so on (Figure 4D).

Nematodes lifespan is known to be regulated by various classical signaling pathways, including insulin/insulin-like growth factor (IIS) pathway, sirtuin 1 signaling pathway, target of rapamycin (TOR) signaling pathway, mitochondrial related genes, and dietary restriction related genes [58]. The KEGG pathway annotation revealed that genes implicated in mTOR pathway and pyruvate metabolism were most significantly altered following Reb A supplementation (Figure 4E). TOR is a serine/threonine kinase that regulates growth, development, and behavior by modifying protein synthesis, autophagy, and various cellular processes in response to nutrient changes [59].

Therefore, we further examined the expression of genes implicated in CeTOR signaling pathway using qPCR, such as *daf-15*, *rict-1*, and *let-363*. The expressions of *daf-15*, which encodes Raptor in TORC1 expression, *clk-2* and *ife-1* were significantly reduced in Reb A treatment groups (Figure 4F). The main upstream signaling pathways of CeTOR includes AMPK signaling pathway, the PI3K/Akt signaling pathway, and MAPK signaling pathway. mRNA levels of *Age-1*, *pdk-1*, and *akt-2* along the PI3K/Akt signaling pathway, which promotes the activation of CeTOR signaling pathway, were significantly reduced in worms treated with Reb A (Figure 4G). Inhibition of the TOR signaling pathway was shown to prolong lifespan in worms, which also mediated larval development, lipid storage, mRNA translation, and autophagy [60]. Indeed, we found that the expression of autophagy-related genes *unc-51*, *pha-4,* and *lgg-1* were upregulated, and the expression of *daf-16*, which encodes DAF-16/FOXO transcription factor, was also significantly increased in Reb A-treated worms. The expression of genes related to stress resistance regulation, such as *mtl-1*, *sod-2*, *sod-3*, and *gcs-1*, were significantly also upregulated in Reb A-treated worms (Figure 4H). Thus, the qPCR results corroborated transcriptomics results and suggest that Reb A treatment prolongs the lifespan of nematodes by inhibiting CeTOR. However, whether Reb A supplementation can bring about phenotype rescue in CeTOR pathway gene mutants awaits further verification.

### 3.5. Reb A Supplementation Lowers Lipid Storage in C. elegans

**Figure 5:**
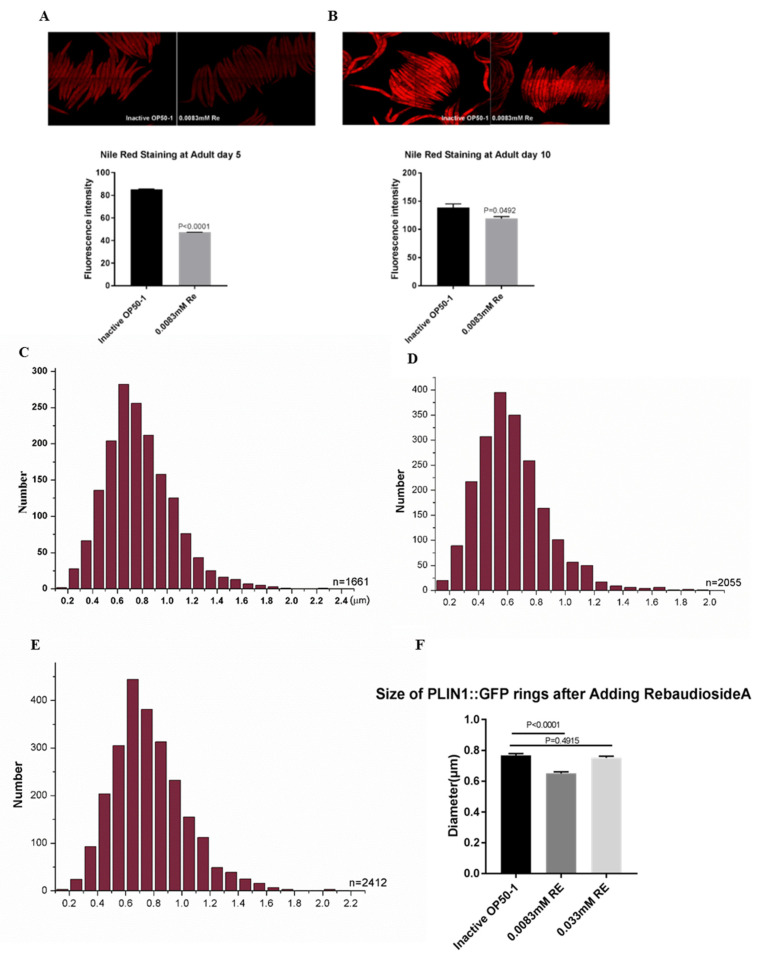
Reb A supplementation lowers lipid storage in *C. elegans*. (**A**) Nile red staining in 5-day-old adults from the 0.0083 mM Reb A and control groups; (**B**) Nile red staining in 10-day-old adults from the 0.0083 mM Reb A treatment and control groups. Statistical significance was calculated by unpaired two-tailed *t* test; (**C**) Distribution of lipid droplet diameters in 1st *xdEx1001* strain in control group; (**D**) Distribution of lipid droplet diameter of 1st *xdEx1001* strain in 0.0083 mM Reb A treatment group; (**E**) The adult lipid droplet diameter distribution of 1st *xdEx1001* strain in 0.033 mM Reb A treatment group; (**F**) Quantification of the size of PLIN1:GFP rings in Reb A treatment groups relative to the control group. Statistical significance was calculated by ANOVA with post hoc Dunnet’s test.

Inhibition of the TOR signaling pathway also leads to upregulation of *lipl-4* expression and enhanced lipolysis [61]. In the current study, the qPCR results showed that both the expressions of lipid desaturase-related genes *fat-5* and lipolysis-related gene *lips-17* were significantly increased in the Reb A groups (Figure 4H). Thus, we examined whether Reb A treatment modified lipid accumulation in *C. elegans*. We further investigated overall lipid storage via NR staining and lipid droplet morphology using the PLIN1:GFP (P*daf-22*-PLIN1:GFP) strain. Consistent with a previous report [20], Reb A reduced lipid storage, in both 5-day-old and 10-day-old adult worms (Figure 5A,B). The expression of *Drosophila* PLIN1:GFP in *C. elegans* labels intestinal lipid droplets under DAF-22 promoter P*daf-22*-PLIN1:GFP. After hybridization of PLIN1:GFP (P*daf-22*-PLIN1:GFP) transgenic strain with wild type N2 nematodes for two generations, fluorescence-labeled worms were selected for expansion. We found that nematodes supplemented with Reb A at 0.0083 mM possess smaller lipid droplets than both the control group and the 0.033 mM Reb A supplementation group (Figure 5C,E). Quantitative P*daf-22*-PLIN1:GFP ring size revealed lipid droplets in worms treated with 0.0083 mM Reb A were significantly reduced in size as compared with those treated with higher concentration of Reb A at 0.033 mM (Figure 5F). Thus, 0.0083 mM Reb A can lower lipid storage in *C. elegans*. Our result is consistent with previous findings that Reb A treatment reduces hepatic TG in mice with non-alcoholic fatty liver disease [20].

### 3.6. Reb A Alters Lipid Metabolism in C. elegans

**Figure 6:**
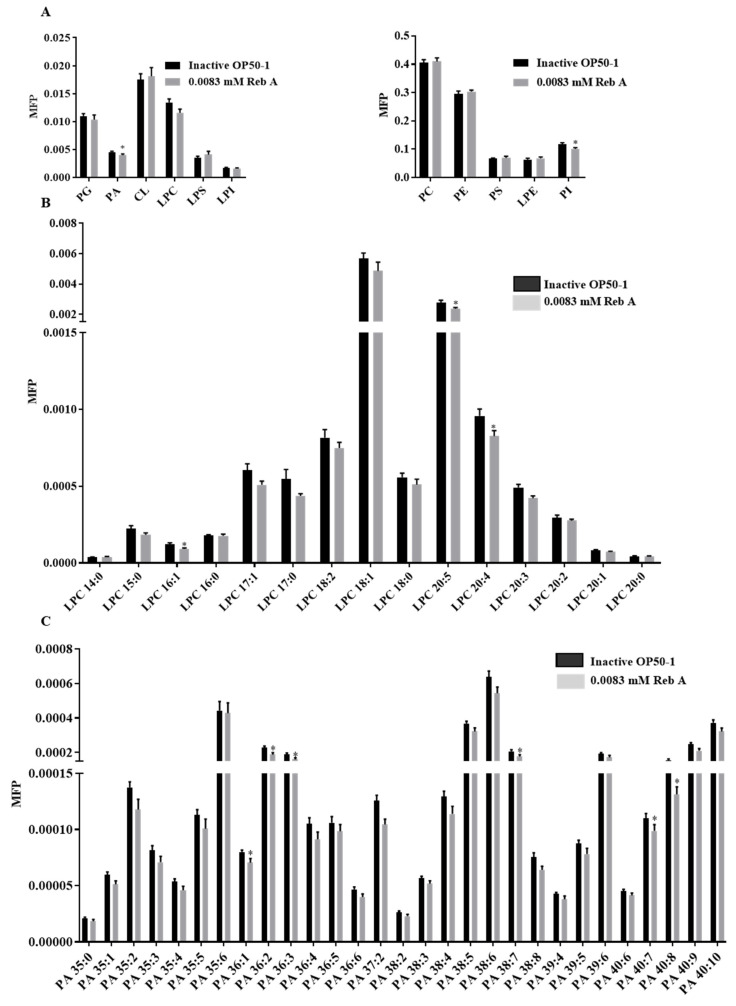

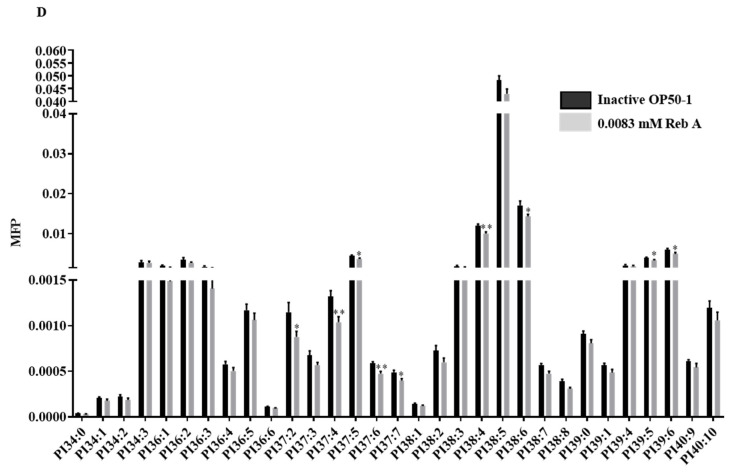
Reb A affects alters lipid metabolism of *C. elegans*. (**A**) The content of phosphatidylcholines (PGs), phosphatidic acids (PAs), cardiolipins (CLs), lysophosphatidylcholines (LPCs), LPS, lysophospholipinositols (LPIs), phosphatidylcholines (PCs), phosphatidylethanolamines (PEs), phosphatidylserines (PSs), lysophosphatidylethanolamines (LPEs), and phosphatidylinositiols (PIs) in the 10-day-old adults were analyzed by lipidomics, presented as molar fractions normalized to total polar lipids (MFP); (**B**) Profiles of LPCs in 10-day-old adult worms; (**C**) Profiles of PAs in 10-day-old adult worms; (**D**) Profiles of PIs in 10-day-old adult worms. Statistical significance was calculated by unpaired two-tailed *t* test. ** *p* < 0.001 and * *p* < 0.05.

Effects of Reb A treatment on lipid storage prompted us to investigate fine changes in the lipidome of Reb A-treated worms. We quantitated phospholipid profiles of the 10-day-old adults after supplementation with Reb A. Changes in the lipid classes of phosphatidylcholines (PCs), phosphatidylethanolamines (PEs), phosphatidylserines (PSs), PIs, PAs, and phosphatidylglycerols (PGs), cardiolipins (CLs), lysophosphatidylethanolamines (LPEs), lysophosphatidylserines (LPSs), lysophospholipinositols (LPIs), and LPCs were illustrated as barplots (Figure 6A). Lipidomics revealed that PAs and PIs were significantly reduced in worms treated with 0.0083 mM Reb A relative to the control (Figure 6A), i.e., LPC-16:1, LPC-20:4, and LPC-20:5 were significantly reduced (Figure 6B), and PA-36:1, PA-36:2, PA-36:3, PA-38:7, PA-40:7, and PA-40:8 were significantly decreased (Figure 6C). As for PIs, mostly polyunsaturated PIs including PI-37:4, PI-37:5, PI-37:6, PI-37:7, PI-38:4, PI-38:6, PI-39:5, and PI-39:6 were significantly reduced in Reb A-treated worms (Figure 6D). Therefore, Reb A alters the phospholipid profiles of *C. elegans*. How Reb A affects membrane phospholipid composition and its possible connection with perturbed neutral lipid storage (i.e., triacylglycerols TAGs that constitute the bulk of lipid droplets) in *C. elegans*, however, warrants further investigation in future studies. In this light, a recent study demonstrated that enhanced de novo biosynthesis of PCs along the TAG-DAG-PC axis underlies the drastic reduction in circulating neutral lipids such as TAGs in GCK-MODY patients [62]. Reductions in PAs upon Reb A treatment may, thus, indicate a reduced supply of lipid intermediates for downstream biosynthesis of neutral lipids, while lowered PIs may indicate perturbations in the PI3K signaling pathway, respectively. High-coverage lipidomics that encompass various forms of fatty acyl derivatives may help to reveal the relevant mechanisms underlying perturbed lipid metabolism and lipid droplet morphology in Reb A-treated worms [63,64], which denotes a meaningful future direction for in-depth interrogation on the working mechanism of Reb A.

## 4. Conclusions

In this paper, we systematically investigated the potential physiological effects of Reb A supplementation on *C. elegans*. We found that Reb A treatment is beneficial towards the lifespan and healthspan of worms, and also improves their associated lipid profiles i.e., lowering neutral lipid accumulation. We showed that the average lifespan and maximum lifespan of nematodes treated with 0.0083 mM Reb A were significantly prolonged, with the maintenance of a younger physiological state. Treatment with Reb A also significantly reduces ROS level and enhances resistance to acute oxidative stress in nematodes.

Transcriptome analysis uncovered DEGs enriched in mTOR signaling pathway in Reb A-treated worms. The qPCR analysis further confirmed that the expression of genes related to the TOR and PI3K/Akt signaling pathways were inhibited, while autophagy-related genes were elevated upon Reb A treatment. Therefore, Reb A may activate autophagy by inhibiting TOR and PI3K/Akt signaling pathways at the molecular level. Finally, we found that lipid storage and levels of PAs and polyunsaturated PIs were significantly reduced in nematodes treated with Reb A. In summary, our study has systematically shown that Reb A, a natural non-nutritive sweetener, prolongs lifespan, enhances oxidative stress resistance, and improves lipid metabolism in vivo in the model organism *C. elegans*, which serves as a ground for future studies exploring the potential medicinal and beneficial effects of Reb A as a replacement for caloric sugars in human foods and beverages.

## Data Availability

Data are available upon reasonable request to the corresponding author.

## Supplementary Materials

The following are available online at https://www.mdpi.com/2076-3921/10/2/262/s1, Figure S1: NNSs pumping rate in *C. elegans*, Figure S2: Different concentration of Reb A effect on lifespan in *C. elegans*.
