# Supplementary material for: Rebaudioside A Enhances Resistance to Oxidative Stress and Extends Lifespan and Healthspan in Caenorhabditis elegans
Source: Antioxidants (Basel). 2021 Feb 8;10(2):262. doi: 10.3390/antiox10020262 (PMC7915623; doi:10.3390/antiox10020262)
Supplement: Supplementary file 1 [file antioxidants-10-00262-s001.pdf]

antioxidants

Supplement Material: Rebaudioside A Enhances Resistance to Oxidative Stress and Extends Lifespan and Healthspan in *Caenorhabditis elegans*

Pan Li [1,2], Zehua Wang [1,2], Sin Man Lam [1,3,*] and Guanghou Shui [1,2,*]

[1] State Key Laboratory of Molecular Developmental Biology, Institute of Genetics and Developmental Biology, Chinese Academy of Sciences, Beijing 100101, China; lipan17@mails.ucas.ac.cn (P.L.); wangzh2014@genetics.ac.cn (Z.W.)
[2] University of Chinese Academy of Sciences, Beijing 100049, China
[3] LipidALL Technologies Company Limited, Changzhou 213022, Jiangsu Province, China
[*] Correspondence: smlam@genetics.ac.cn (S.M.L.); ghshui@genetics.ac.cn (G.S.); Tel.: +86-(10)-6480-6670 (S.M.L.); +86-(10)-6480-7781 (G.S.)

In addition to natural NNS Reb A, we also tested pharyngeal pumping rate after treating worms with artificial NNS neotame and low-caloric oligosaccharides raffinose. Neotame- (1 mM, 2 mM, 4 mM) and raffinose- (3 mM, 4 mM) treated worms showed significantly lower pharyngeal pumping rate in the 5th day adults relative to control groups (Supplement Figure S1A), whereas for the 10th day adults, 4 mM neotame and 4 mM raffinose supplementation significantly reduced pharyngeal pumping rate (Supplement Figure S1B). Therefore, Reb A was subsequently chosen for further investigation of its potential anti-aging effect on *C. elegans.*

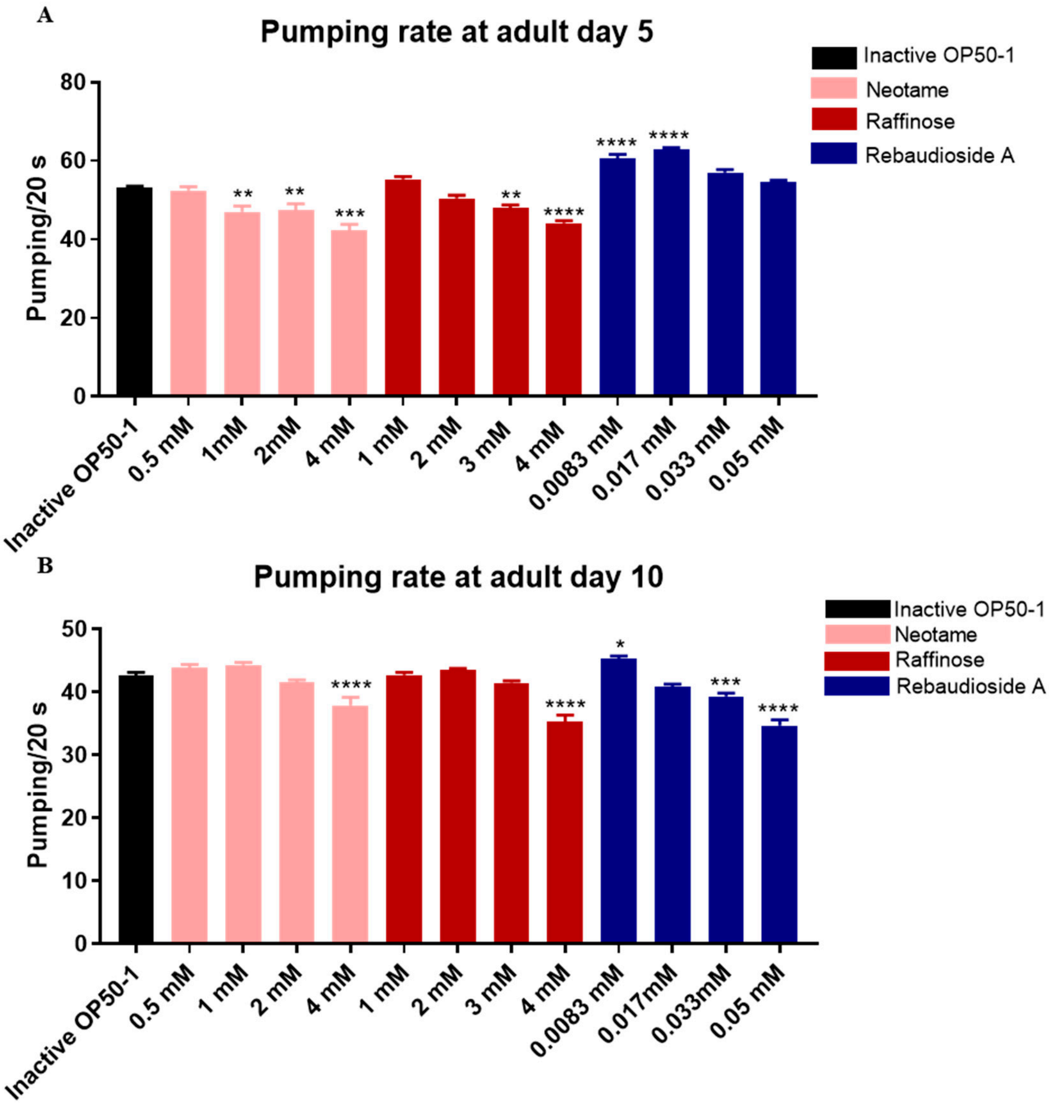

Supplement Figure S1. NNSs pumping rate in *C. elegans*. (**A**) Pumping rate in worms (wild-type N2 strain) at the 5th day of adulthood on control NGM, as well as NGM supplemented with designated concentrations of neotame, raffinose and Reb A, respectively. (**B**) Pumping rate in worms (wild-type N2 strain) at the 10th day of adulthood in on control NGM and NGM supplemented with designated concentrations of neotame, raffinose and Reb A. One-way ANOVA with post-hoc Dunnet's test was used for evaluation of statistical significance.

We had set five different concentrations to investigate the effect of Reb A on lifespan extension. While all concentrations tested extended lifespan in *C. elegans* to varying extents, treatment with 0.0083 mM Reb A brought about the greatest expansion in lifespan. Therefore, we adopted the treatment concentration of Reb A at 0.0083 mM for all subsequent experiments.

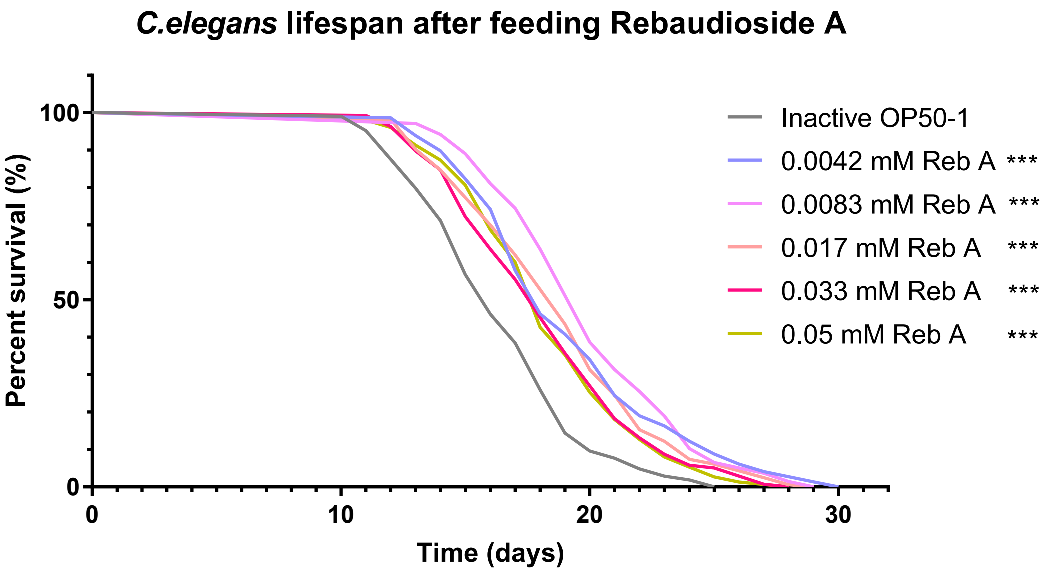

Supplement Figure S2. The effects of Reb A supplementation on lifespan in *C. elegans*. Survival plot and lifespan assay comparing worms grown on control NGM and that supplemented with varying concentrations of Reb A (0.0042 mM–0.05 mM). Statistical significance was calculated by Log-rank (Mantel-Cox) test, *** $p < 0.001$.
